# The Potential of Induced Pluripotent Stem Cells to Treat and Model Alzheimer's Disease

**DOI:** 10.1155/2021/5511630

**Published:** 2021-05-26

**Authors:** Joseph M. Schulz

**Affiliations:** Burnett School of Biomedical Sciences, College of Medicine, University of Central Florida, Orlando FL 32816, USA

## Abstract

An estimated 6.2 million Americans aged 65 or older are currently living with Alzheimer's disease (AD), a neurodegenerative disease that disrupts an individual's ability to function independently through the degeneration of key regions in the brain, including but not limited to the hippocampus, the prefrontal cortex, and the motor cortex. The cause of this degeneration is not known, but research has found two proteins that undergo posttranslational modifications: tau, a protein concentrated in the axons of neurons, and amyloid precursor protein (APP), a protein concentrated near the synapse. Through mechanisms that have yet to be elucidated, the accumulation of these two proteins in their abnormal aggregate forms leads to the neurodegeneration that is characteristic of AD. Until the invention of induced pluripotent stem cells (iPSCs) in 2006, the bulk of research was carried out using transgenic animal models that offered little promise in their ability to translate well from benchtop to bedside, creating a bottleneck in the development of therapeutics. However, with iPSC, patient-specific cell cultures can be utilized to create models based on human cells. These human cells have the potential to avoid issues in translatability that have plagued animal models by providing researchers with a model that closely resembles and mimics the neurons found in humans. By using human iPSC technology, researchers can create more accurate models of AD *ex vivo* while also focusing on regenerative medicine using iPSC *in vivo*. The following review focuses on the current uses of iPSC and how they have the potential to regenerate damaged neuronal tissue, in the hopes that these technologies can assist in getting through the bottleneck of AD therapeutic research.

## 1. Introduction

A common theme in current neurodegenerative biomedical research is collaboration and using an interdisciplinary approach to solve problems. These problems can be genetic, molecular, or cellular, so determining the root cause of the neurodegeneration is useful in helping create an effective treatment against the uncovered pathology. To accomplish this, a new field of biomedical research has emerged: Translational Medicine (TM). TM integrates basic sciences and clinical medicine with the aim of optimizing the preventative measures and patient care, as well as increasing the turnout and expediting the process of turning appropriate biological discoveries into efficacious treatments or appropriate medical devices [1].

The appropriate application of TM will be useful in overcoming the bottleneck associated with (1) the identification and validation of appropriate biomarkers for early or preclinical diagnosis as well as monitoring the clinical progression of the diseases, (2) promoting the innovative clinical technologies, such as neuroimaging, stem cell technology, and nanotechnology, and (3) expediting the development of novel drug candidates by using appropriate organisms to model clinical conditions [1]. These organisms include, but are not limited to, invertebrates such as *Caenorhabditis elegans*, *Drosophila melanogaster*, and *Danio rerio* (zebrafish) [2–4] and mammalian vertebrates, such as rodents or mice [5, 6]. Although important molecular cascades have been uncovered using these model organisms, these KO/KD transgenic organisms do not translate well to the clinical setting [7]. The limitations associated with animal models include extrapolating rare, well-understood genetic variants of a disease to treating a more common, less-understood sporadic form of the same disease, artificial overexpression of proteins in transgenic/AAV-mediated models that does not return to basal levels with the inclusion of a knock-in variant, the shorter lifespan of models that does not allow for the complete development of the pathogenesis in age-related neurodegenerative diseases, and the lack of complex brain development in these organisms the does not allow for interpreting behavioral deficits that are characteristic to human neurodegenerative diseases [5]. For a complete review on different animal models and their shortcomings, refer to Dawson et al. or Drummond et al. [5, 8]; see Dubey et al. for a complete review on cellular models [9].

One approach researchers attempted to overcome the hurdles associated with animal models was the use of pluripotent stem cells (PSC), such as murine embryonic stem cells (ESC), which are undifferentiated cells with self-renewal capabilities and the potential to differentiate into any cell type of the body, providing researchers an opportunity to model human diseases with human cells [10]. Prior to 2007, the only type of PSC being used in research was ESC, and these were limited in scope due to the ethical questions surrounding the use of ESC. In 2006, Takahashi and Yamanaka generated iPSC from mouse somatic cell lines and then later repeated this experiment with human cells, thus creating hiPSCs [11, 12]. These new cells behave similarly to ESC, in which they can differentiate into any cell types of the body.

However, without the ethical limitations associated with ESC, iPSC biotechnology gives a larger community of researchers access to technology that can be of great aid to biomedical and clinical research. Given this great leap in science, questions remain about the limitations that PSC possess, including what these cells can be utilized for. In this review, PSC will be broken down into the different types of stem cells, as well as the application that these stem cells may have for neurodegenerative diseases, such as Alzheimer's disease (AD). Through the use of stem cells, diseases can be modeled, therapeutics tested for efficacy, and the potential to regenerate lost tissue tested using translational models.

## 2. Pluripotent Stem Cells

When a sperm cell and an ovum fuse in the fallopian tube, fertilization begins, and a zygote is formed. As the zygote divides, it forms a ball of cells known as a blastocyst. This blastocyst contains an outer cell mass (OCM) and inner cell mass (ICM). The OCM forms the trophoblast, which differentiates into an inner layer called the cytotrophoblast and an outer layer called the syncytiotrophoblast, which protects the lacunae by secreting human chorionic gonadotropin (HcG) [13]. Together, these two layers form the placenta around the developing embryo. The ICM forms the embryoblast, the precursor to all the cells of the human body. Embryoblast cells are short-lived and begin their differentiation into more specialized cells as implantation occurs. Initially, they form a bilaminar disc, the epiblast, which gives rise to the mesoderm, endoderm, and ectoderm, and the hypoblast, which gives rise to the yolk sac and chorion [14]. If implantation is prevented, the ICM will not differentiate and these cells, derived from the assisted reproductive technology (ART) programs, can be cultured and studied in research laboratories.

It has been nearly 40 years since ESC were first isolated from the ICM of the developing mouse blastocyst and grown *in vitro* [15, 16]. However, it was not until 1998 when the first derivation of human ESC was reported in the literature [17]. ESC have been shown to contribute to the endoderm, ectoderm, and mesoderm, as well as the germ line, when incorporated into chimeras with intact embryos [18–29]. *In vitro*, ESC can be indefinitely propagated in the undifferentiated state by growth in the presence of the leukemia inhibitory factor (LIF) and/or layer of murine embryonic fibroblasts (MEF), yet they retain the ability to differentiate to all mature somatic phenotypes when induced by the correct set of transcriptional factors [30–32]. The initial isolation in 1981 ushered in a new era of developmental biology by providing researchers with an appropriate model to study processes of early cellular programming and differentiation. When ESC were derived from humans in 1998, regenerative medicine and tissue engineering in humans finally became a real possibility. ESC have the potential to be used in the treatment of a great number of diseases in which the body is not naturally able to fully repair organ damage or dysfunction properly, thus leading to life-threatening complications.

The ability to differentiate into different organs means that the safety and efficacy of drugs can be tested on more reliable human-cell-based models [33–36]. For example, patients with an inherited mutation in the *HERG* gene develop long QT syndrome, a cardiac repolarization disorder that predisposes affected individuals to arrhythmia which can lead to sudden fainting or even death [37]. Certain small-molecule therapeutics has the potential to block the potassium channel, which prevents the potassium from leaving the cell and can quickly lead to myocardial infraction in certain individuals; therefore, screening drugs early on to check their inhibition against these channels is crucial in the development of efficacious drugs. Myocardial cells that express these *HERG* channels can be cultured, and different drugs can be screened against them to test the cytotoxicity [38]. This approach saves resources by preventing researchers and large pharmaceutical companies from optimizing therapies that will not translate to the clinical setting.

However, a big wrench was thrown in ESC research when President Bush banned federal funding for research on newly created ESC lines and specified that research prior to August 9^th^, 2001, would still be eligible for funding [39]. This ban on funding limited the ability for researchers to investigate ethnic differences in cell populations and limited the ability for researchers to investigate new diseases [39]. The lines that remained were of poor therapeutic value due to inferior conditions in which the cells were cultured and maintained [40]. Luckily, when the new administration took over, President Obama signed an executive order that reversed the previous decision and allowed the federal funding of hundreds of viable stem cell lines that were previously restricted [39]. This funded new groups to investigate the previously unavailable lines, specifically unused embryos from ART fertility clinics, but it did not allow for funding embryos created specifically for research purposes or derived from other sources [39]. This limit on funding means researchers have to utilize different methods to investigate diseases, such as animal models, which have their own swath of investigative issues, or the now revolutionary iPSC, which allows for the investigation of almost any human ailment using human-derived somatic cells.

Retrovirus-mediated transduction gives researchers the ability to transform single-stranded RNA into double-stranded DNA that can be incorporated into the DNA of dividing host cells. This technique has enabled researchers to infect target cells and reprogram their genetic makeup, forcing them to exhibit a specific biochemical response [41–45]. Retrovirus-mediated transduction of human fibroblasts with four transcriptional factors (Oct-3/4, Sox2, KLF4, and MYC), all of which are expressed in ESC, could induce the fibroblast into an iPSC [12]. The ectopic expression of these four transcription factors reverses the previous shutdown that occurred when the cell became specialized during development. OCT4 and SOX2 induce the pluripotent gene pathway and enhance the expression of NANOG, a critical transcriptional factor present in the morula-stage embryos, ICM, and the epiblast, but not the primordial germ cells (PGC), intraembryonic mesoderm, and extraembryonic endoderm [46]. A deficiency in NANOG triggers the differentiation of ESC to the extraembryonic endoderm lineage, suggesting that this DNA-binding protein acts in part by transcriptionally repressing key regulators of this alternative tissue fate [47]. NANOG-null embryos were unable to support the formation of the epiblast and subsequent ESC, producing an endodermal only derivatives [48]. MYC is not necessary for the pluripotency exhibited by the iPSCs. Instead, it is important to regulate chromatin structure to facilitate cellular reprogamming [49]. KLF4 interacts with pluripotency network proteins, including SOX2 and OCT4, and also inhibits cell death [47]. In normal cellular development, OCT4 is zygotically expressed in the four to eight cell stages and is continued to be expressed in the ICM of the blastocyst [50]. The downregulation of OCT4 leads a zone of trophoblastic specification in the outer edge cells of the morula [51]. This demonstrates that OCT4 acts as a negative regulator of differentiation in the trophectoderm and a critical regulator of the pluripotent capabilities of the ICM [51–54]. This further demonstrated failure of OCT4-null embryos to form the ICM, instead differentiating into trophoectoderm [50]. SOX2 mutants demonstrated limited differential capabilities, leaving only trophoblast giant cells and extraembryonic ectoderm [47]. These mutants allow the formation of the blastocyst cavity; however, it lacks the ICM. In murine SOX2-knockout (KO) models, failure of the ICM means ESC are not developed and the mice are not viable past early embryonic development; however, wild-type ESC injection into the SOX2 mutant can rescue expression and prevent epiblast defects [55, 56]. KLF4 promotes cell survival by suppressing the p53-dependent apoptotic pathway by directly inhibiting TP53 and suppressing BAX expression [57, 58]. Coupled together, these four transcription factors are capable of reprogramming almost any specialized cell.

The main benefit of using iPSC is the avoidance of using an oocyte, especially for use in patient-specific therapies because the patient would be able to donate their own cells for autotransplantation [47]. This also avoids issues associated with partial major histocompatibility (MHC) matches because the surface antigens from donors would match the patients and avoid elucidating an immune response. This is one of the benefits of using a patient's own cells to treat a patient-specific ailment.

An additional benefits of using fibroblast-derived iPSC are that they can be used to differentiate into different types of neuronal cells, such as forebrain acetylcholine neurons, dopaminergic progenitor cells (substantia nigra pars compacta (SN_PC_)), Purkinje cells, hippocampal cells, and striatal cells, managing to exhibit electrical responses characteristic of neuronal firing [59–68]. This potential for successful reprogramming might be possible because the nervous system and ectoderm originate from the same embryonic tissue, the neuroectoderm [69]. These iPSCs can be transplanted into the region of interest (ROI) in the brain tissue of transgenic animal models, and the effects on different cognitive abilities can be observed, such as learning, memory, arousal, motor function, and motivational response [70]. However, as previously stated, higher-level cognitive abilities that are characteristic of certain neurodegenerative diseases are difficult to study, even with the addition of iPSC technology in transgenic animal models. Nonetheless, iPSCs are being utilized and studied for their potential for patient-specific clinic treatments in different neurodegenerative diseases.

## 3. Alzheimer's Disease

### 3.1. Economic Impact of AD

In 2010, roughly 5 million individuals aged 65 years or older in the United States were diagnosed with AD, the leading cause of dementia [71]. By 2050, AD is predicted to affect just under 14 million individuals, almost tripling in impact in just 40 years [71]. Not only does AD have an economic impact on society but it also costs families 11-70 hours per week in care, doing tasks such as feeding, bathing, and caring for their affected family member [72]. The costs associated with care were just under $19,000 in 1998, owing to the costs associated with caregiving time and a caregiver's lost earnings [72]. Owing to inflation, that same amount would cost just over $30,000 in 2021. In 2015, it was estimated that approximately 18.1 billion hours of assistance was provided by roughly 16 million Americans, estimated to cost $221 billion dollars [73]. As the disease progresses, the family is not able to provide the adequate care that is necessary for the patient and they are then placed in an assisted living facility. These facilities alone have a median cost of $4,051 per month, or $48,612 per year [74]. The economic impact this disease will have on society will continue to grow until improved therapeutics and treatments arise.

### 3.2. Pathology of AD

Psychologically, AD is characterized by early progressive anterograde amnesia, followed by slow progressive retrograde amnesia. These symptoms coincide with impairments in executive functions and other behavioral disturbances, which include paranoia, agitation, and impairment in spatial and temporal memory [5, 75]. Biologically, AD has three hallmark pathologies: insoluble extracellular senile plaques comprised of amyloid beta (A*β*), insoluble intracellular neurofibrillary tangles (NFT) comprised of hyperphosphorylated tau, and degeneration in the hippocampal formation and cerebral cortex [76–80]. The amyloid precursor protein (APP) is a type I transmembrane protein that is highly conserved in vertebrates and consists of three homologues: APP, amyloid precursor-like protein 1 (APLP-1), and amyloid precursor-like protein 2 (APLP-2) [81]. For autosomal dominant early-onset Alzheimer's disease (EOAD), mutations in the APP, presenilin 1 (PSEN1), or presenilin 2 (PSEN2) gene sequence are a major risk factor, while the APOE4 allele is a major risk factor for late-onset Alzheimer's disease (LOAD) [82]. Excess A*β* is believed to contribute to the dysfunction seen in AD by leading to the formation of senile plaques; however, amyloid plaques have been found in other diseases, including vascular dementia, Lewy body dementia, and Parkinson's disease with dementia, as well as in the brain of aged individuals without any cognitive deficits [83–86]. The presence of A*β* in otherwise healthy individuals demonstrates that A*β* may have an intrinsic property in the normal physiology of neurons that is not yet understood.

Briefly, APP can be cleaved by three enzymes: *α*-secretase, *β*-secretase, and *γ*-secretase. PSEN1/2 is a component of *γ*-secretase, and it is a combination of these three enzymes cleaving the carboxyl end of APP that results in the formation of different protein fragments. For example, cleavage of *α*-secretase followed by *γ*-secretase results in soluble amyloid precursor protein *α* (sAPP*α*) and P3 [87]. This cleavage is hypothesized to be beneficial to neurons against oxygen-glucose deprivation and cellular excitotoxicity by inhibiting calcium currents and increasing potassium currents which effectively stabilized the resting membrane potential of neurons [88, 89]. sAPP*α* was also shown to promote neurite outgrowth, synaptogenesis, and cell adhesion [90, 91]. The formation of sAPP*α* prevents the formation of A*β* because *α*-secretase cleaves the APP protein at a site within 10 amino acids of the location *β*-secretase would cleave [87]. A*β* is formed when *β*-secretase cleaves the APP protein to form soluble amyloid precursor protein *β* (sAPP*β*), followed by cleavage by *γ*-secretase, resulting in insoluble A*β*. This insoluble A*β* has the potential to induce conformational changes in soluble APP fragments, resulting in the senile plaques that are seen postmortem.

Intracellularly, tau is a member of the microtubule-associated proteins (MAPs) that stabilize neuronal microtubules (MTs) for their role in the development of cell processes, establishment of cell polarity, and axonal intracellular transport, both anterograde and retrograde [77]. A single *tau* gene on chromosome 17 codes for the tau protein that has 6 isoforms due to alternative splicing [92]. KO of the gene in *Drosophila* was not detrimental to the behavior, survival, or neuronal function [93], possibly because other MAPs can be substituted to stabilize MTs and the subsequent wild-type (WT) function is not affected. Tau mRNA is transported to the proximal axon from the cell body where translation occurs, and a gradient exists of tau protein, with the highest concentration found in the proximal axon, decreasing the more distal tau is from the cell body [94, 95]. Tau itself is an intrinsically disordered protein (IDP) that adopts a conformation that allows it to stabilize the MTs without being relegated to a single, rigid conformation [96, 97]. Its ability to adopt multiple conformations depends on posttranslational modification activity from both kinases and phosphatases. Tau kinases are classified as proline-directed (PDPK) and non-proline-directed protein kinases (NPDPK) [98]. One example of PDPK is glycogen synthase kinase 3 (GSK-3), which phosphorylates numerous sites in the tau protein, as well as in murine models overexpressing GSK-3 [96, 99, 100]. In tau phosphatases, the most significant enzyme is protein phosphatase 2A (PP2A) which accounts for more than 70% of the total posttranslational modification activity found in the human brain [101, 102]. Excess activity in the kinases or decreased activity in the phosphatases at specific phosphorylation sites can result in hyperphosphorylated tau (p-tau) [96, 103]. Many of the abnormal phosphorylation sites are at Ser-Pro or Thr-Pro motifs [77], which might explain the difficulty phosphatase enzymes encounter when removing a phosphate group at the Ser or Thr amino acid. Pro contains a rigid 5-membered nitrogen ring that forms a peptide bond with the adjacent amino acid's carbonyl group, via a condensation reaction. The hyperphosphorylation of tau at PDPK sites may induce a conformational change in the normally fluid tau at the Pro site residue, possibly changing its conformation from a *cis* to a *trans*-conformation to reduce any steric hindrances that the additional of an electronegative phosphate group might have on the peptide bond between the two residues. The phosphate group can also form salt bridges with neighboring arginine groups [104], another example of a posttranslational modification that potentially impacts PP2A activity and ability to remove phosphate groups.

A high concentration of p-tau consequently results in the depolymerization of MTs when it loses its IDP properties and adopts a rigid conformation [104]. The depolymerization of the MTs results in the reduction of length and size of the axons and increases the concentration p-tau in the intracellular matrix. Eventually, p-tau aggregates to form paired helical filaments (PHF), which bundle to form the intracellular NFTs seen in the postmortem pathology of AD [105]. PHF are not characteristic of only AD and have been characterized in frontotemporal dementia (FTD) linked to a V337M *MAPT* mutation as well as D252V and G389_I392del mutations [106, 107]. These mutations and subsequent phenotypes demonstrate that MAPs play an important role in regulating intracellular activity in neurons found in various regions of not only the hippocampus but also the cerebral cortex.

The third and final pathological hallmark of AD is the degeneration of neurons in the hippocampal formation and cerebral cortex, findings that are studied with neuropsychological examination but only confirmed upon autopsy. Neuropsychological exams are able to assess the global cognitive ability, memory, and executive function of the patient [108] but offer little in the ability to monitor the atrophy of the actual brain tissue. Ideally, researchers want to monitor the atrophy of the brain in a living person to test the effects of potential therapeutics against degeneration, and so they employ an array of biomarkers or imaging techniques [109–111]. However, in order to develop effective biomarkers, effective drugs that target AD pathology are needed to test the efficaciousness of the biomarkers. The best treatments that exist are acetyl cholinesterase inhibitors (AChEI) like donepezil and rivastigmine, which slow down the symptoms associated with AD by blocking the uptake of acetylcholine (ACh) into the postsynaptic neuron [112–115]. It has also been shown that donepezil may play a role in suppressing inflammatory responses in the brain [115, 116] and that this inhibition of the inflammatory system may slow down any damage caused by microglia in the hippocampus and cerebral cortex. Currently, it is not known what makes the hippocampus vulnerable to atrophy; however, a number of neurochemical and vascular alterations, such as deviations in the levels of glucocorticoids, serotonin, glutamate, and their subsequent receptors, have been implicated [64, 117]. Understanding what causes the atrophy in this region at the cellular level will elicit the biochemical processes that link A*β* pathology to NFT pathology, and this knowledge will enable the next generation of therapeutics to be developed that target the pathology instead of the symptoms.

## 4. Stem Cells in AD

### 4.1. Modeling

#### 4.1.1. Genetics

iPSCs derived from peripheral blood mononuclear cells (PBMC) and fibroblasts have the potential to revolutionize the drug discovery process by providing researchers with a model that has the potential to usurp animal models as the model of choice among researchers through a translatable model derived directly from the cells of patients who have been diagnosed with neurodegenerative diseases [118, 119]. However, waiting for patients to develop symptoms associated with AD means that the pathology has developed past the point of preventative medicine and enters the realm of improving the quality of life. Therefore, genetic models of AD are utilized in the creation of effective iPSC models (Table 1). Cells derived from a patient with a double mutation in the APP gene (KM670/671NL) increased the total levels of A*β*, while cells derived from a patient with a duplicated APP gene revealed higher levels of A*β* (1–4) and p-tau (Thr231) and increased activity in GSK3B [120, 121]. As a side note, there is a gene mutation in *APP* (A673T) that was shown to be protective against cognitive decline by decreasing levels of sAPP*β* [122]. iPSCs were generated from a patient with this mutation [123] and are being investigated to uncover the cellular processes that the increased polarity from this mutation might have on the shape, function, and environment of the APP protein.

Patients with trisomy 21 have an extra copy of *APP*, found on the 21^st^ chromosome, which is associated with elevated levels of A*β*, an overaccumulation of which has been shown to lead to AD dementia in patients with Down syndrome [124–126]. iPSCs derived from mesenchymal stem cells (MSC) in amniotic fluid from individuals with trisomy 21 demonstrate the ability to model the pathology in AD, such as elevated levels of A*β* and increased levels of p-tau [127]. As individuals with Down syndrome represent a population that is at risk of developing AD, the iPSC cell lines can be used to screen different therapeutics for their ability to reduce the levels of p-tau and A*β*.

Mutations of *PSEN1/2*, the catalytic component of *γ*-secretase, have been linked to familial Alzheimer's disease (fAD). Patients with fAD have mutations in *PSEN1* (A246E) and *PSEN2* (N141I) [70]. A separate *PSEN1* exon 9 deletion (PSEN1*δ*9) produced mutant astrocytes that altered the calcium signaling activity of healthy neurons when AD astrocytes generated from iPSC from PSEN1*δ*9 donor cells were cocultured with healthy neurons [128]. Toxic A*β*42 secretion was seen in neurons derived from *PSEN1* mutation donor cells [129], demonstrating the potential that fAD iPSC models possess for modeling AD. However, abnormal issues with *γ*-secretase represent a small portion of patients diagnosed with AD, so these models might not be the most translatable.

*Microtubule-associated protein tau* (*MAPT*) gene mutations are the most prevalent cause of familial frontotemporal dementia (fFTD), a condition linked to mutations on chromosome 17 (p.A152T), which has also been implicated in AD and Parkinson's disease with dementia [130]. The mutation leads to an additional phosphorylation site that has the potential to form salt bridges with nearby amino acids. If post-translational modifications of this mutant tau by phosphorylation changes the 3D conformation to a more stable, rigid conformation, then understanding how this mechanism works is the key to reversing and developing therapeutics that prevents the formation of p-tau and its aggregates. iPSC-derived neurons were generated from individuals carrying the p.A152T variant, and it was established that upregulation of p-tau was coupled with enhanced stress-inducible markers and cell vulnerability to proteotoxic, excitotoxic, and mitochondrial stressors, which were rescued by CRISPR/Cas9-mediated targeting of tau or by pharmacological activation of autophagy [131]. With iPSCs producing mutant tau, it becomes possible to elucidate and uncover the cellular mechanisms that underpin protein misfolding in tauopathies, mainly by studying the effects seeding with p-tau has on microtubule formation in these derived neurons. A separate study used zinc finger nucleases (ZFNs) to introduce two *MAPT* mutations in healthy donor iPSC: an IVS10+16 mutant shown to increase the inclusion of exon 10 and a P301S point mutation in exon 10 [132]. The former mutation was selected for its potency to fasten the inclusion *MAPT* exon 10 while the latter mutation was chosen to generate an aggressive fFTD model [132]. This model would provide researchers with a genetic model of tauopathy that can be used in conjunction with other models to study the effects therapeutics have on p-tau without the presence of A*β*.

The largest population of patients diagnosed belong to the LOAD group, and of this group, the most prevalent genetic risk factor is *APOE4*, which is linked to the sporadic form of the disease, sporadic Alzheimer's disease (sAD). Apolipoprotein E (apoE) is produced primarily by astrocytes in the CNS as a carrier of cholesterol and other lipids that support the membrane, synaptic integrity, and injury repair [133, 134]. In one experiment, ApoE4 secreted by glia cells stimulated A*β* formation by binding with APOER found on the extracellular surface of iPSC-derived neurons, initiating a noncanonical cascade that results in the upregulation of Mitogen-Activated Protein (MAP) kinase kinase kinase, also known as Dual Leucine zipper-bearing Kinase (DLK), an attractive candidate for neuronal signaling because it has been implicated in axonal regeneration, synaptogenesis, and neurodegeneration [134–136]. Mixed-lineage kinase (MLK) MKK7 was previously shown to be found in the same cellular compartment as DLK and phosphorylation target, and overexpression of DLK led to increased levels of phosphorylated MKK7 (pMKK7) and subsequent levels of phosphorylated ERK1/2 MAP kinase [134]. This cellular cascade led to the upregulation of APP independent of APLP1 and APLP2, by activating a DLK-dependent MAP kinase signaling pathway that induces cFos phosphorylation which stimulates AP-1 and enhances APP synthesis via a direct effect on the *APP* gene promoter [134].

A separate study derived neurons, astrocytes, and microglia-like cells from isogenic *APOE3* and *APOE4 iPSC* lines to examine the cellular differences exhibited between cells from donors with different alleles [137]. These results showed that *APOE4* astrocytes and microglia were less efficient in the uptake and clearance of A*β* compared to *APOE3* astrocytes, but it did not determine if ApoE is necessary for the clearance of A*β* from the extracellular matrix as reduced *APOE4* mRNA and protein levels were seen in iPSC-derived astrocytes, indicating the effect is specific to astrocytes [137]. The APOE4 variant was shown to regulate the expression of numerous lipid metabolism and transport genes, leading to the accumulation of cholesterol in the intracellular and extracellular space in the glial cell cultures [137].

In 2D cultures without A*β*, *APOE4* microglia exhibited fewer and shorter processes than *APOE3* microglia; however, after embedding in 3D neuronal cultures that produced A*β*, the same cells had longer processes than their APOE3 counterparts, consistent with impairment in the ability of APOE4 microglia-like cells to respond effectively to A*β* in the environment [137–139]. One of the upregulated immune genes seen in the microglia-like cells was *IRF8*, an immune-related gene that has been shown to induce transcription of many other immune-related genes, transforming the resting microglia into a reactive state [140]. The expression of TREM2 and its signaling adaptor TYROBP, proteins crucial for microglial function and a significant AD risk gene, was positively correlated with the APOE4 genotype [141, 142] and is consistent with recent studies that show increased levels of TREM2 in cerebrospinal fluid of AD patients [143], but further work is needed in order to determine the exact mechanism linking TREM2 and ApoE.

#### 4.1.2. Organoids

Along with diseased neurons, researchers can generate astrocytes, oligodendrocytes, microglia, and the vasculature of the brain in 2D or 3D models in order to examine the cellular dysfunction that arises during the development and interaction of different cell types in AD [10, 144–146]. For a more encompassing and complete review on brain organoid protocols, current advances, and limitations, refer to Papaspyropoulos et al. [147] A scaffold-free 3D model generated from fibroblasts of controls and patients with fAD, the result of a duplication in APP or a PSEN1 mutation, resulted in elevated levels of A*β* and p-tau in organoids from fAD cultures compared to controls [148]. These organoids were treated with a BACE-1 *β*-secretase inhibitor and a *γ*-secretase inhibitor that are well known to inhibit the aggregation of A*β* [149] or a DMSO vehicle that acted as the control. After 60 days of treatment, the particle counts of A*β* had decreased and immunoreactivity of p-tau decreased, signifying a decrease in the concentration of p-tau [148]. These results demonstrate that elevated A*β* levels correlates positively with levels of p-tau and that treatment that decreases A*β* levels subsequently decreases the concentration, potentially via a cellular mechanism that results in the reduction of GSK3*β* activity. By inhibiting *β*-secretase and *γ*-secretase, *α*-secretase and low levels of *γ*-secretase are cleaving APP into sAPP*α* which promotes neuroprotective factors and stimulates neurite outgrowth [91], a process that is mediated by the binding of tau to MTs in the axon [94].

In separate studies, sAPP*α* overexpression led to low levels of GSK3*β* activity and decreased levels of p-tau [121, 150], providing evidence that APP processing might underpin the pathology exhibited in AD through currently intracellular interactions. This hypothesis coincides with the evidence from studies of patients with elevated A*β* levels with no cognitive deficits. We investigated the intracellular matrix and subsequent proteome of the neurons of these types of patients compared to neurons of individuals homozygous for *APOE4* who have been diagnosed with MCI or AD. This comparison experiment could potentially uncover proteins or a signaling cascade that prevents the adverse effects that improper APP processing has on otherwise healthy cells by comparing the relative levels of protein expression between the two populations.

To examine the effects of the immune system on the brain organoid development and maturation, organoids were generated that included both neuronal cells and microglial cells [151]. The microglia, being the resident macrophages of the brain, have sparked interest in recent years as potential targets for immunotherapies that are aimed at reducing the inflammation and subsequent damage caused by the phagocytosis of neurons, both *in vivo* and *ex vivo* [152–155]. Microglia differ from peripheral blood monocytes that derive from myeloblastosis protooncogene and transcription factor- (MYB) dependent HSC in the bone marrow by originating from MYB-independent yolk sac-derived fetal macrophages that invade the brain around embryonic day 31 until the closure of the blood-brain barrier where they proliferate locally in the brain and are not replaced by peripheral macrophages in the body [156].

Since microglia have a distinct embryonic origin, microglia-derived iPSC from HSC will resemble the monocyte-derived cells found in the brain that have a morphology similar to that of resident microglia, but its function and transcriptome differ significantly from those of the native microglia [157]. A more in-depth analysis of microglia protocols is presented by Haenseler and Rajendran [156], the main takeaway being that a near-authentic microglia model should mimic the microglial ontogeny and neuronal environment by differentiating in an MYB-independent manner to yolk sac-derived fetal macrophages that are allowed to invade a neuronal environment where they can mature and adopt the healthy, resident microglia phenotype and avoid creating a “microglia-like” cell that does not imitate the interactions seen in neurodegenerative diseases. Coculturing microglia and neurons will not only improve preclinical models but also improve translatability from benchtop to bedside by improving drug screening. One such screen could be to examine synaptotoxicity of neurons with fluorescently tagged synapses (using synapsin I, synapsin II, or synaptophysin as markers) [158] and microglia containing an activation marker, such as allograft inflammation factor 1(AIF-1) [159]. First, conditions that induce microglia-driven synaptotoxicity would need to be identified, either *in vivo* or *ex vivo*. These conditions could be a prion protein that induces an inflammation response in the microglia or a pathogen that activates the microglia into phagocytizing the otherwise healthy neurons. Once a coculture system exists, small molecules can be assayed and screened to find potential hits, molecules that are capable of interrupting the interaction between activated microglia and neurons and preventing the induced synaptotoxicity and subsequent neuronal loss.

### 4.2. Reconstruction

iPSCs not only have the potential to be used for modeling diseases *ex vivo*, implanting autologous gene-edited iPSC-derived cells into patients opens up Pandora's box of new therapeutic potential. iPSC-derived microglia have been shown to integrate successfully into the brains of murine models [160–162]. Transplantation of human long-term neuroepithelial-like stem (It-NES) cell-derived cortical neurons at two months into stroke injured rats produced from iPSC improved neurological deficits and established both afferent and efferent morphological and functional connections with host cortical neurons at 5 months, as demonstrated by the presence of cortical phenotype cells with pyramidal morphology and the presence of the cortex-specific marker TBR1 and lack of tumorigenesis [163–165]. At 6 months after transplantation into rats with ischemic lesions in the cerebral cortex, host neurons in the contralateral somatosensory cortex received monosynaptic inputs from grafted neurons [165]. Immunoelectron microscopy demonstrated the myelination of the graft-derived axons in the corpus callosum, and their terminals formed excitatory glutamatergic synapses on host cortical neurons [165]. Optogenetic inhibition of the It-NES cells and the subsequent loss of motor function in the murine model demonstrated their involvement in the regulation of the stroke-induced animals' behavior [163, 164]. These experiments demonstrate that transplantation of hiPSC into a murine model is possible and that the recovery of lost motor function can be achieved in a live murine model.

Taking the previous experiment further, healthy neocortical tissue from the middle temporal gyrus of patients undergoing elective surgery for epilepsy was cocultured with It-NES cells and was shown to form functional afferent and efferent connections with adult human cortical neurons in the slices, evidenced by electron microscopy, rabies virus retrograde monosynaptic tracing, and whole-cell patch clamp recordings [166]. This experiment provides evidence that hiPSC can differentiate into layer-specific functional synaptic networks when implanted onto organotypic cultures. This finding supports the clinical translatability that neuronal replacement with iPSC-derived cells might possess in neurodegenerative diseases by strengthening the functional networks that are damaged due to the loss of tissue.

Furthermore, this grafting, in patients with AD, might ameliorate or even prevent the neurodegeneration seen in the cortex of AD patients. In a human trial of 50 patients living with Parkinson's disease (PD), autologous implantation of stem cells with highly selective arterial catheterization was performed into the posterior region of the circle of Willis and the quality of life (QOL), activities of daily living, depression, and disability were evaluated for two years [167]. No complications arose from this treatment, and improvements in all of the categories were seen in the patients, especially the QOL.

In a separate phase I clinical study, human umbilical cord blood MSC were stereotactically injected into the precuneus, the site where amyloid accumulation is believed to begin, and the hippocampus, the site where NFT aggregation is seen [168]. MSC are unlikely to differentiate into the neurons; however, they potentially secrete cytotropic factors into the brain that could decrease neuroinflammation by reducing total amyloid load and increasing endogenous neurogenesis [169]. The patients received a bilateral injection into the hippocampus or a lateral injection into the right side of the precuneus to compare the change in amyloid burden between the MSC-treated right precuneus region and the untreated left precuneus region. Adverse events were recorded, such as wound pain where the burr hole was created in all of the patients, headache, dizziness, delirium, nausea, and back pain which were noted in a small minority of the patients, but none of these symptoms were considered serious enough to halt the trial. The conclusion of this phase I trial determined that administration of MSC derived from umbilical cord blood into the hippocampus and precuneus was feasible, safe, and well tolerated in patients with mild-moderate AD [168]. One caveat with these results was the lack of a control group to compare these results to. Without a proper control, the efficacy could not be determined; however, further studies should be conducted to determine the clinical benefit of this treatment by comparing experimental MSC results with placebo treatments on a larger cohort.

## 5. Shortcomings

iPSC-derived cells from humans have been investigated for their potential in improving translatability from benchtop to bedside. These cells have the capabilities of modeling diseases, like AD, *ex vivo* and *in vivo. Ex vivo* experiments using hiPSC can be conducted at a faster rate than animal models, allowing for the rapid understanding of the effects of different KO and knock-ins. Behavioral assays on cell cultures cannot be accomplished, but hiPSCs that are xenotransplanted into the brains of murine models were shown to form functional synaptic connections with the native tissue, a finding that was recapitulated in hiPSC cultured with healthy neocortical tissue. Using organotypic slices preserves key cellular elements of the brain, such as glial cells and neurons, as well as morphological and electrophysiological properties that are consistent with pyramidal neurons *in vivo*, and provides a 3D architecture that preserves its synaptic connections and microenvironment [148, 170–172]. However, the use of these slices does not allow for the study of its mechanisms of interaction to be fully elicited due to the absence of components of the vascular and immune systems and the decreased survival of the neurons with long-term culturing [166]. Furthermore, an injury response involving reactive microglial cells and progressive neurodegeneration is seen in resected human tissue [173]. This injury response was a result of the procedure and not a pathological immune response of the grafting.

Brain organoids derived from hiPSC are capable of recapitulating key aspects of the human brain; however, they are not a perfect replica. Therefore, overcoming limitations of the organoid will expand the ability to investigate human brain development and disorders associated with abnormal development. Currently, one of the greatest pitfalls in organoid technology is the small number of current organoids as well as the batch-to-batch variability that arises from a diverse number of protocols being followed by researchers. The eventual establishment of a human brain atlas containing immunohistology data, *in situ* hybridization, and transcriptomics data will give researchers developing and engineering organoids a “gold standard” by which they can compare their lab organoids to the tissue of a “standard” human brain [174]. With a gold standard, organoid engineers will be able to further engineer a “gold standard” organoid to which further organoids that model neurodegenerative diseases can be compared, enabling researchers to test different therapeutics, such as iPSC regeneration treatment or small-molecule drug therapy, on a translatable model.

Considering that the development of iPSC technologies provides an attractive possibility of using differentiated somatic human cells as a platform to model diseases or regenerate tissue, one of the greatest shortcomings is the genomic instability exhibited by iPSCs [47, 132, 175–179]. Whole exome sequencing was done on the human foreskin fibroblast at two different passages to determine if the mutations seen in iPSC are due to stress associated with oncogene expression during reprogramming, and the researchers found that *in vitro* passaging contributed to 7% of the mutations; 19% of the mutations were preexisting and were derived from parental fibroblasts, suggesting that 74% of the mutations were acquired during cellular reprogramming [177]. Structural variations in the chromosome are also seen; the most recurrent are chromosome deletions, which cause a loss of heterozygosity, and duplications of chromosomes [175], which might be advantageous to the growth and survival of the culture, but at the same time, these chromosomal aberrations can confer a completely different phenotype to a cell, potentially creating a teratoma. One example of a beneficial duplication is trisomy 12. Chromosome 12 contains cell cycle-related genes and the pluripotency-associated gene *NANOG* [179]. Duplication of this chromosome has the potential to contribute to the selective advantage of proliferation and reprogramming of iPSC by providing the cell with more NANOG. This additional NANOG might allow the cell to reprogram itself, making this mutation favorable for the reprogramming phase of iPSC and allow for the differentiation to a specific cell type.

Epigenetic genomic imprinting mechanisms, such as histone modification and DNA methylation, function to regulate chromosome architecture and the transcriptional repression of repetitive elements and regulate and repress gene activity during development [180, 181]. DNA methylation modifies CpG dinucleotides and is associated with a transcriptionally repressed state, effectively silencing the gene on either the maternal or the paternal allele [182]. Compared with ESC, iPSC generated from blood, fibroblast, and brain tissue exhibited a much greater tissue-specific epigenetic signature [183], due to incomplete reset of the tissue-specific epigenetic signature to the default embryonic stages during the process of reprogramming. These tissue-specific epigenetic signatures originate during the development of the embryo, at certain stages of somatic cell differentiation and dedifferentiation under tightly regulated gene expression [47]. The genomic instability of iPSC could result from (I) preexisting mutations in parental somatic cells, (II) reprogramming-induced mutations, and (III) mutations that arise during *in vitro* culture [184]. This genomic instability could hamper *in vitro* models of AD because the presence of genomic deletions and amplifications exhibited by the iPSC-derived neurons is suggestive of oncogene-induced DNA replication stress [185]. This replication stress, usually located in the common fragile sites (CFS), has the potential to alter the phenotypes exhibited by iPSC and prevent them from fully exhibiting their differentiated properties that are specific to the cells of interest; this could be caused by aneuploidy, an abnormality in chromosomal number, single-nucleotide variations (SNV), and subchromosomal copy number variation (CNV), all of which have the potential to promote the spontaneous loss of chromosomes [186]. If, for example, a researcher is trying to study APP, a protein coded on chromosome 21 in fibroblast-derived neurons, an abnormality in this chromosome could potentially impact the transcription and subsequent translation of the proteins of interest, resulting in a shift in production that would not be found in normal neuronal conditions, resulting in an *in vitro* experiment that provides results for a mutated phenotype, instead of the desired phenotype.

In addition, genomic instability can alter the ability of iPSC to reconstruct the cellular morphology *in vivo.* One such alteration that can arise involves the tumor suppressor P53 gene [187]. Normally, P53 induces cell cycle arrest, apoptosis, or senescence of the stressed somatic cells to prevent the passage of genetic abnormalities; in iPSC, p53 is silenced to allow the reprogramming transcriptional factors to revert the somatic cell into a cell that can be differentiated [188]. Given the importance p53 has on maintaining genetic stability, silencing this gene and then transplanting the cells for *in vivo* culture could result in the formation of a teratoma at the site of implantation.

One way to overcome the hurdle posed by transferring epigenetic markers to iPSC would be through the use of a nuclear transfer to an unnucleated oocyte (ntESC) [189]. These ntESC provide genetically identical and immunologically compatible stem cells for individual somatic cell donors; however, this process is arduous and inefficient. However, the lack of tissue-specific epigenetic memory seen in ntESC provides evidence that the ooplasm contains additional factors needed to competently erase tissue-specific epigenetic memory, and research is currently being undertaken to determine these additional factors. One study attempted to reverse the incomplete reprogramming status of iPSC after iPSC nuclear transfer to an enucleated oocyte [190]. They found that iPSC-nt-ESC showed even worse developmental potential compared with the original iPSC, indicating that aberrant gene expression pattern established during iPSC derivation cannot be reset by nuclear transfer [190], potentially because of genetic aberrations acquired during iPSC formation [175]. This experiment demonstrated that faulty gene expressions that existed previously in iPSC cannot be reset by nuclear transfer, nor can it reverse developmental deficiencies characteristic of iPSC.

Identification of differentially methylated regions (DMRs) between iPSC and ESC is an important starting point. High-resolution DNA methylation analysis identifies DMRs in iPSC and compares them with the findings in ESC and somatic cells, allowing the researchers to determine the source of the epigenetic change. Another technique used to abrogate the epigenetic differences exhibited by iPSC derived from different origins is continuous passaging [191]. They found that the RNA expression profile of 12 different iPSC lines was notably different at the fourth passage; however, by the 16^th^ passage, the expression profile of the iPSC was reduced from between 500 and 2000 differentially expressed genes to less than 50 in the late passage cultures [191]. Extensive *in vitro* passaging has the ability to reduce the variability seen in iPSC derived from different origins. However, the use of earlier passages of iPSC is favored in a therapeutic application to avoid genetic and epigenetic changes that arise during the extended culturing process. A different approach would be to use a chromatin-modifying compound that enables a DNA demethylation agent, such as 5-aza-cytidine [192], to remove the methylation that is tissue specific, restoring the ability to differentiate to various tissue lineages [193]. However, this approach does not improve the pluripotency and potentially damages other regions of DNA that are susceptible to modifications.

Site-specific targeting of hiPSC is also important in regenerating damaged CNS tissue, so more research needs to be conducted that bridges the gap between biomarkers of the central nervous system (CNS) that differentiate neural lineages into the specific tissue [194] and the ability of hiPSC to differentiate into these specific brain regions without (1) generating an immune response, (2) forming cancerous teratomas *in vivo*, or (3) forming non-site-specific tissue, while also (4) regaining lost brain function, both physical (electrophysiological, histological) and psychological, and (5) being reproducible. These five pillars need to be followed if neurodegenerative diseases, like AD, are hoped to have any treatment that improves the quality of life while also treating the neurodegeneration that precedes the psychological symptoms of the disease. These five pillars can be applied to any regenerative treatment that is aimed at successfully treating damaged tissue in the body, substituting item (4) for whatever organ the researcher aims to study, such as the liver, heart, or kidney and focusing on regaining its lost molecular functions.

## 6. Conclusion

To improve the QOL of patients diagnosed with Alzheimer's disease, the next generation of therapeutics needs to be developed. However, in order to develop effective therapeutics, model organisms that recapitulate the pathology of the disease need to be studied in order to ascertain the mechanisms that lead to neurodegeneration. The past 50 years have relied heavily on transgenic animal models that do not translate well to the phenotype's characteristic of the disease, relying heavily on silencing gene expression or overexpression of proteins to elicit a pathological response. These methods, although effective at inducing protein misfolding or aggregation, do not accurately represent the cascade of events that underlies the pathology seen in sporadic Alzheimer's disease, the leading cause of AD in patients. To better understand the pathology that underlies neurodegeneration seen in sAD patients, induced pluripotent stem cell models generated from the patient should be utilized to not only model the degeneration, thus elucidating the mechanisms that underlie the abnormal protein responses in sAD, but also reconstruct damaged or degenerated neural tissue. Once the kinks have been hammered out of iPSC, they have the potential to revolutionize the way we model and treat diseases of the body.

## Figures and Tables

**Table 1:** Current genetic iPSC models of Alzheimer's disease.

Gene	Model/mutation	Phenotype	References
APP	iPSC/KM670/671NL	Increased levels of A*β* p-tau (Thr231) GSK3B activity ↑ Neurodegeneration	[120, 121]
	iPSC/A673T	Decreases levels of sAPP*β* Neurodegeneration	[123]
	MSC/trisomy 21	A*β* expression ↑ p-Tau expression ↑ Neurodegeneration	[127]
PSEN1	iPSC/PSEN1*δ*9	Mutant astrocytes Disrupted Ca2+ signaling in healthy neurons Toxic A*β* secretion Neurodegeneration	[128, 129]
MAPT	iPSC/IVS10+16, P301S	4R:3R tau expression increased Perturbations in Ca^2+^ burst frequency Reduced lysosomal acidity Tau oligomerization Neurodegeneration	[132]
APOE	iPSC/APOER (*R* = 2, 3, or 4)	Allelic expression of APOE influences APP transcription through an abnormal kinase cascade APOE4 astrocytes and microglia exhibited a decrease in A*β* clearance Accumulation of cholesterol in the intra- and extracellular matrices A*β* expression led to the activation of microglia; however, the length of processes was allelic dependent	[134, 137, 139]
